# Polymeric Sorbent Sheets Coupled to Direct Analysis in Real Time Mass Spectrometry for Trace-Level Volatile Analysis—A Multi-Vineyard Evaluation Study

**DOI:** 10.3390/foods9040409

**Published:** 2020-04-02

**Authors:** Madeleine Y. Bee-DiGregorio, Hui Feng, Bruce S. Pan, Nick K. Dokoozlian, Gavin L. Sacks

**Affiliations:** 1 Department of Food Science, Cornell University, 411 Tower Rd, Ithaca, NY 14853, USA; myb8@cornell.edu; 2 E&J Gallo Winery, 600 Yosemite Blvd, Modesto, CA 95354, USA; Hui.Feng@ejgallo.com (H.F.); Bruce.Pan@ejgallo.com (B.S.P.); Nick.Dokoozlian@ejgallo.com (N.K.D.)

**Keywords:** high-throughput analysis, volatile analysis, grape aroma, DART-MS, SPME

## Abstract

Etched polymeric sorbent sheets (solid-phase mesh-enhanced sorption from headspace (SPMESH) sheets) were recently described as an alternative to solid-phase microextraction (SPME) for rapid, parallel, multi-sample extraction and pre-concentration of headspace volatiles. In this report, a workflow was evaluated based on SPMESH sheet extraction followed by direct analysis in real time-mass spectrometry (DART-MS) using grape samples harvested from multiple commercial vineyards at different maturities. SPMESH sheet-DART-MS(-MS) was performed on two grape-derived odorants related to wine quality: 3-isobutyl-2-methoxypyrazine (IBMP) in Cabernet Sauvignon and Merlot grape homogenate (*n* = 86 samples) and linalool in Muscat-type grape juice samples (*n* = 18 samples). As part of the optimization process, an MS-MS method was developed for IBMP and an equilibration procedure prior to extraction was established for homogenate samples. Following optimization, we achieved good correlation between SPMESH sheet-DART-MS and SPME-GC-MS for both IBMP (range by GC-MS = < 2 ng/L to 28 ng/L, *R*^2^ = 0.70) and linalool (range by GC-MS = 135 to 415 μg/L, *R*^2^ = 0.66). The results indicate SPMESH sheet-DART-MS is suitable for rapid measurements of trace level volatiles in grapes.

## 1. Introduction

Considerable variation can be found in the aroma of commercial wines, even when produced from the same grape variety [1,2,3]. This variation in wine aroma (and thus wine chemistry) can arise from differences in winemaking practices [4] but can also reflect differences in concentration of odorants or their precursors in the original grape material [5,6,7]. Several grape volatiles are reported to be useful markers for eventual wine sensory qualities. For example, 3-isobutyl-2-methoxypyrazine (IBMP, “green pepper aroma”) is a potent odorant found above its sensory threshold in Bordeaux-type grapes such as Merlot and Cabernet Sauvignon. IBMP concentration in grapes is reported to be predictive of resulting red wine quality [8]. Monoterpenes such as linalool (“honeysuckle, floral aroma”) are the key odorants primarily responsible for the aroma of Muscat-type grapes such as Muscat Blanc and Muscat of Alexandria [9] and is therefore a marker for the intensity of Muscat wine aroma [10].

However, one challenge with using odorants as part of routine quality assessments of grapes is that these compounds have low sensory thresholds and are present at trace concentrations, e.g., the odor threshold for IBMP in red wine is ~10 ng/L [11] and, for linalool in white wine is 25 μg/L [12]. Trace odorants in grapes and other foodstuffs are typically measured by gas chromatography–mass spectrometry (GC-MS), generally after a preconcentration and extraction step such as headspace solid-phase microextraction (SPME). In a typical SPME analysis, volatiles are isolated onto a fiber coated with an ab/adsorbent material, and the fiber subsequently desorbed into the GC injector port [13]. SPME-GC-MS is sensitive, robust, and easily automated, but is also throughput-limited (30–60 min/sample) due to the time necessary for GC separation and/or SPME extraction [14].

Several recent reviews have highlighted the advantages of using thin-film (TF)-SPME devices in place of classic fiber-based SPME geometries [15,16,17]. TF-SPME is readily coupled with ambient-ionization mass spectrometry approaches that allow for direct sample introduction into an MS without a chromatographic step, like direct analysis in real time (DART-MS). For example, TF-SPME devices consisting of a biocompatible polymer coated onto stainless steel meshes were successfully interfaced with DART-MS for analysis of pesticides, with low μg/L detection limits and analysis times < 1 min/sample [15]. These reports of TF-SPME-DART-MS focused on applications for non-volatiles, likely because the adsorbent particles employed are less appropriate for volatile extraction.

We have recently reported on an alternative TF-SPME approach, “SPMESH” (solid phase mesh enhanced sorption from headspace), which can be combined with DART-MS for rapid analysis of trace volatiles [18,19,20]. In its original form, SPMESH devices consisted of commercial OpenSpot™ cards, in which the stainless steel mesh of the cards was coated with an absorbent layer (polydimethylsiloxane (PDMS), PDMS-divinylbenzene (DVB) [19,20]. Extractions were performed by positioning the coated mesh in the headspace of a sample vial. Using these “one-shot” SPMESH devices, we evaluated the use of SPMESH-DART-MS against SPME-GC-MS for measurement of IBMP and linalool in real grape samples [19]. However, this work had several shortcomings, primarily that it could not be readily automated in the one-shot format and therefore method throughput was limited. Additionally, in our evaluation of the one-shot approach, a poor correlation was observed at native, low ng/L-level concentrations of IBMP [19].

In subsequent work, we produced SPMESH devices by laser-etching a fine mesh pattern (0.5 × 0.5 mm) into thin PDMS sheets (“SPMESH sheets”). These sheets were of similar dimensions to standard multi-well plates, which allowed for parallel headspace extraction and preconcentration of volatiles from multiple samples [18]. After SPMESH sheet extraction, volatiles were desorbed and analyzed by DART-MS. By using an automated X-Z positioning stage, we were able to quantify IBMP (200–1000 ng/L) in 24 samples in 17 min. Good accuracy was also observed for recovery spikes of IBMP and linalool into grape macerate, and the sheets could be baked-out for reuse. However, the detection limits (39 ng/L for IBMP and 48 μg/L for linalool) were relatively high as compared to the published sensory thresholds for these compounds in wine (10 ng/L and 25 μg/L, respectively). Additionally, we did not evaluate the SPMESH sheet-DART-MS approach using samples containing only native concentrations of target analytes, as opposed to spiked analytes.

**Figure 1:**
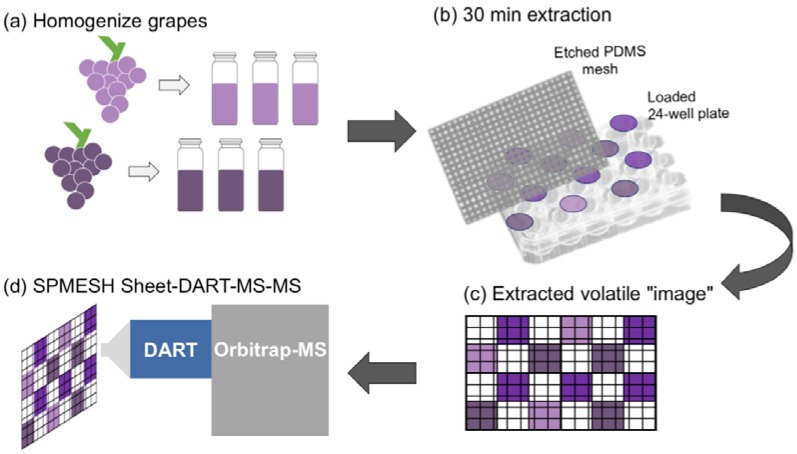
Schematic overview of sample preparation, SPMESH (solid phase mesh enhanced sorption from headspace) extraction and DART (Direct Analysis in Real Time)-Orbitrap-MS analysis.

In this work, we report on an optimization of a SPMESH sheet-DART-MS method for analysis of two volatiles in grapes (IBMP and linalool; Figure 1). Specifically, we show that off-line sample prep and MS-MS improves the reproducibility of this SPMESH sheet approach and lowers the methodological detection limits below the odorants’ sensory thresholds. For the first time, we evaluated the SPMESH sheet-DART-MS method of native odorants in grapes by comparing results against SPME-GC-MS using multiple grape samples from commercial vineyards during harvest.

## 2. Materials and Methods

### 2.1. Materials

Deep well, 10 mL, 24-well plates were purchased from VWR (Randor, PA, USA). PDMS sheets (CultureWell Silicone Sheet, 0.25 mm thick, 13 cm × 18 cm) were purchased from Grace Bio-Labs (Bend, OR, USA). Laser-etching of PDMS sheets was performed at the Cornell Nanoscale Facility (Ithaca, NY, USA). Spacers and clamps for the SPMESH sheet extraction devices were machined at the Cornell University LASSP machine shop (Ithaca, NY, USA). Twenty mL amber SPME vials and caps were purchased from MilliporeSigma (Burlington, MA, USA). Sodium chloride (NaCl), methanol and ethanol were purchased from VWR (Radnor, PA). IBMP and linalool were purchased from MilliporeSigma (Burlington, MA), and their internal standards *d*_3_-isobutyl-2-methoxypyrazine (*d*_3_-IBMP) and *d*_3_-linalool were purchased from C/D/N Isotopes (Point-Claire, Quebec). *Vitis vinifera* grapes (Pinot noir *n* = 1 (for calibration), Cabernet Sauvignon *n* = 69, Merlot *n* = 43, Chardonnay *n* = 1 (for calibration), Muscat Blanc *n* = 5, Muscat of Alexandria *n* = 12, Malvasia Bianca *n* = 1 and Symphony *n* = 1) were collected by E & J Gallo Winery (Modesto, CA, USA) from vineyards in Modesto, Lodi, Lake County, Fresno, Madera, Alexander Valley, Paso Robles, Napa Valley, Dry Creek Valley and Central Coast AVAs throughout July to September of the 2019 harvest, at commercial maturity (18 to 25 °Brix).

### 2.2. SPMESH Sheet-DART-MS

The general approach for SPMESH sheet extraction from a multi-well plate followed by DART-MS analyses was adapted from a previous report (Figure 1) [18]. Briefly, the SPMESH sheet was baked-out at 250 °C for 15 min in a GC oven immediately prior to extraction. Samples and/or calibration standards were loaded into a 24-well plate. A Teflon gasket was laid over the plate, followed by a stainless-steel spacer, the SPMESH sheet, a second stainless steel spacer and finally, a cover. The assembly was then clamped tightly and incubated at 50 °C for 30 min, with agitation (230 RPM). After extraction, spacers, gaskets, well plates and clamps were rinsed with ethanol and allowed to dry prior to the next use.

The SPMESH sheet was then removed from the assembly and transferred to a 3DS-XZ automated positioning stage in front of a DART standardized voltage and pressure (SVP) ion source (Ionsense Inc.—Saugus, MA, USA). The DART source was coupled to a Q Exactive mass spectrometer (Thermo Fisher Scientific—Waltham, MA, USA) via a Vapur^TM^ interface (Ionsense, Inc.). The DART source was operated in transmission mode and movement of the positioning stage in front of the MS inlet was controlled by the DART web-based software. The DART desorption temperature was set to 450 °C, with a scan speed of 0.5 mm/s, based on previous work [18].

Both the DART and MS were operated in positive ion mode with high purity He as the carrier gas. The Orbitrap was operated with 140.000 scan resolution, 1 microscan, 250 ms maximum injection time and automatic gain control (AGC) of 1 × 10^6^ IBMP and *d*_3_-IBMP were measured by MS-MS using the parallel reaction monitoring (PRM) mode at 5 ppm mass tolerance: 167 > 124.0632 *m/z* for IBMP, 170 > 127.0818 *m/z* for *d*_3_-IBMP, over a mass range 124–128 *m/z.* Transitions were identified previously [20], but exact mass was confirmed on the Orbitrap. Collision energy (CE) was set at 25 eV. Linalool and *d*_3_-linalool were measured using extracted ions from full scans over a mass range of 137–141 *m/z* at 5 ppm mass tolerance: 137.1326 *m/z* for linalool, 140.1512 *m/z* for *d_3_*-linalool. For MS/MS, the following transitions were measured: 137 > 67.0549 *m/z* for linalool and 140 > 70.0736 *m/z* for *d*_3_-linalool over a mass range 67–71 *m/z*; transitions were identified by dropping standard solutions with a Pasteur pipette in front of the DART source while running a product ion scan on the Orbitrap.

### 2.3. SPME-GC-MS Conditions

An Agilent 7890B GC system coupled to an HP 5973 MSD (Agilent—Santa Clara, CA, USA) equipped with the Gerstel Multipurpose Sampler (Gerstel Inc.—Linthicum, MD, USA) autosampler was used for evaluation experiments. Grape samples were prepared as juice (for linalool analysis) or homogenate (for IBMP analysis) as described in “Evaluation of SPMESH sheet-DART-MS in comparison SPME-GC-MS.” below, except that the 7 mL sample was combined with 10 μL internal standard solution, 20 μL sodium dodecylsulfate (SDS) to disrupt enzymatic activity and 3 mL saturated NaCl solution to increase volatility before loading into 20 mL amber autosampler vials. A 1 cm, 50/30 µm PDMS/DVB/CAR SPME fiber (Supelco—Bellefonte, PA, USA) was pre-incubated at 70 °C for 60 s, incubated in the sample headspace at 70 °C for 1080 s and desorbed for 500 s starting at 62 °C, increasing to 225 °C at 90 °C/min in splitless mode for 2 min into a split/splitless injection port. A 30 m × 0.25 mm × 0.50 µm DB-WAX UI column (Agilent—Santa Clara, CA, USA) with ultra-high purity helium carrier gas at a constant flow rate of 1.0 mL/min, initial pressure of 6.86 psi, purge flow of 50 mL/min (after 2 min of splitless) and total flow of 54 mL/min. The MS ion source temperature was 230 °C and interface temperature was 180 °C. The GC oven temperature ramp was as follows: start at 35 °C, increase to 42 °C at 6 °C/min, increase to 75 °C at 11 °C/min, increase to 135 °C at 6 °C/min, increase to 195 °C at 11 °C/min and increase to 240 °C at 120 °C/min for a final runtime of 24.4 min. The MS was operated in SIM mode, and the following ions were monitored: 121, 93, and 136 *m/z* for linalool, 124 and 151 *m/z* for IBMP, 124 and 74 *m/z* for *d_3_*-linalool and 154 and 127 *m/z* for *d*_3_-IBMP.

### 2.4. Sample Preparation for Determining Figures of Merit

Stock solutions of unlabeled standards and deuterated internal standards were prepared in methanol such that spiking 150 µL of stock solution into 15 mL of juice or grape macerate resulted in the following calibration concentrations: 400, 200, 100, 50, 25, 12.5, 6.25 ng/L IBMP with 100 ng/L *d*_3_-IBMP; 400, 200, 100, 50, 25 µg/L linalool with 100 µg/L *d*_3_-linalool. Calibration standards were prepared by spiking into Chardonnay juice (for linalool) or Pinot noir homogenate (for IBMP) that contained no detectable IBMP or linalool by SPME-GC-MS. The juice and homogenate used for calibration standards was prepared as described in “Evaluation of SPMESH sheet-DART-MS in comparison to SPME-GC-MS”. Each calibration level was analyzed in triplicate. Quantification was performed by matrix-matched calibration curves, in which the standards were spiked into grape juice or homogenate with undetectable concentrations of the target analyte (Chardonnay or Pinot noir). Because IBMP and linalool have low volatility at room temperature as defined by Henry’s Law coefficients, losses due to volatilization during sample transfer were expected to be limited. Limits of detection were calculated from calibration curves based on 3 × signal-to-noise (S:N) ratio; limits of quantification were based on 10 × S:N.

### 2.5. Optimization of Sample Preparation for Homogenate and Juice Samples by SPMESH Sheet-DART-MS

In initial optimization experiments, IBMP was quantified by both SPMESH sheet-DART-MS and SPME-GC-MS in homogenate prepared from Cabernet Sauvignon and Merlot samples (*n* = 26, “Optimization of Grape Homogenate Preparation for IBMP Analysis”). A subset of four samples was used to evaluate several different sample preparation techniques: homogenate, homogenate with added NaCl and supernatant; all of which were compared to SPME-GC-MS results for real samples. Elevated equilibration temperatures were also tested.

Grape macerate was prepared as follows: de-stemmed grapes were homogenized in a blender and stored at −20 °C until use. Fifteen grams homogenate and 150 µL internal standard solution were vortexed in a 20 mL amber SPME vial. Vials were equilibrated in an Innova 4080 Incubator Shaker (New Brunswick Scientific—Edison, NJ, USA) initially at 50 °C (*n* = 24, “Optimization of Grape Homogenate Preparation for IBMP Analysis”) after optimization (*n* = 86, “Optimization of grape homogenate preparation for IBMP analysis”) at 70 °C for 1 h while agitating at 230 RPM [21]. The contents of each vial were transferred into individual wells (approximately 5 g per well, in triplicate) in the 24-well plate prior to headspace extraction.

To test NaCl addition, 4.5 mL of saturated NaCl solution was combined with 10.5 mL homogenate and 150 µL internal standard solution to generate 15 mL total sample in a 20 mL amber SPME vial. Vials were equilibrated in an Innova 4080 Incubator Shaker (New Brunswick Scientific—Edison, NJ, USA) at 70 °C for 1 h while agitating at 230 RPM [21]. The contents of each vial were transferred into individual wells (approximately 5 mL per well, in triplicate) in the 24-well plate prior to headspace extraction.

Supernatant was generated from red grape homogenate by centrifuging at 4000 RPM in a Beckmann Spinchron-15 for 15 min and poured off the solids. Fifteen mL supernatant and 150 µL internal standard solution were vortexed in a 20 mL amber SPME vial. Vials were equilibrated in an Innova 4080 Incubator Shaker (New Brunswick Scientific—Edison, NJ, USA) at 50 °C for 30 min while agitating at 230 RPM [21]. The contents of each vial were transferred into individual wells (approximately 5 mL per well, in triplicate) in the 24-well plate prior to headspace extraction.

To test higher temperature equilibration conditions, Pinot noir grapes (containing no detectable IBMP by SPME-GC-MS, also used in calibration curves) were homogenized and spiked with internal standard solution as described above. Equilibration was performed at 70 °C for 1 h, prior to analysis by DART.

All extractions and analyses were conducted as described in “SPMESH sheet-DART-MS”.

### 2.6. Evaluation of SPMESH Sheet-DART-MS in Comparison to SPME-GC-MS

Linalool was quantified by both SPMESH sheet-DART-MS and SPME-GC-MS in juice prepared from Muscat-type grapes (*n* = 18). IBMP was quantified by both SPMESH sheet-DART-MS and SPME-GC-MS in homogenate prepared from Cabernet Sauvignon and Merlot samples (total *n* = 110: *n* = 24 under initial conditions, *n* = 86 by the optimized conditions, both described in “Optimization of sample preparation for homogenate and juice samples analyzed by SPMESH-sheet-DART-MS”). SPME-GC-MS samples were prepared as described in “SPME-GC-MS Conditions”. All samples were analyzed in triplicate. The two methods were compared by linear regression.

For preparation of juice samples from white grapes, no optimization was needed and grapes were initially crushed in a stand mixer, with any remaining intact berries ruptured by light pressing. The juice was strained through cheesecloth to remove grape skins. Juice was analyzed the day it was received or stored at −20 °C until use. In a 20 mL amber SPME vial, 15 mL juice and 150 µL internal standard solution were combined and vortexed thoroughly. After spiking standards, vials were equilibrated in an Innova 4080 Incubator Shaker (New Brunswick Scientific—Edison, NJ, USA) at 50 °C for 30 min while agitating at 230 RPM. The contents of each vial were transferred into individual wells (approximately 5 mL per well, in triplicate) in the multi-well plate prior to headspace extraction.

For statistical analyses, undetectable values were set to the square root of the LOD [22] (2 ng/L for IBMP by DART, 1.4 ng/L for IBMP by SPME, 4.89 µg/L for linalool by DART, 1 µg/L for linalool by SPME). Two outliers were removed from IBMP initial evaluation data, determined by quantile range outliers in JMP, with tail quantile of 0.1 and Q of 3; leaving 24 of 26 in the initial evaluation data and 86 samples included in the final evaluation. All statistical tests were performed using JMP Pro, Version 14.0 (SAS Institute Inc., Cary, NC, USA, 1989–2007).

## 3. Results and Discussion

### 3.1. Calibration Curves and Figures of Merit

**Figure 2:**
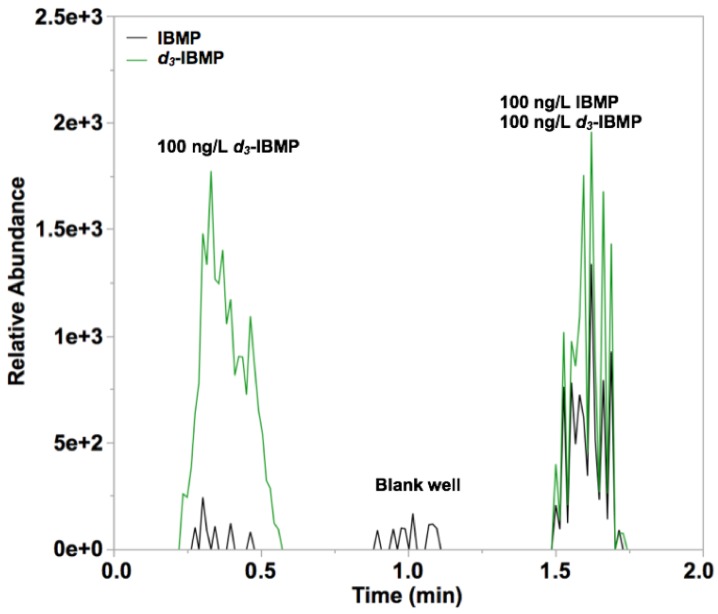
DART-Orbitrap-MS chronograms of extracted ions for IBMP (2-isobutyl 3-methoxypyrazine) 167 > 124.0632 *m*/*z* and *d*_3_-IBMP 170 > 127.0818 *m*/*z* in Pinot noir homogenate (contains no native IBMP); (**left**) spiked with only internal standard, (**middle**) blank well and (**right**) spiked with both standard and internal standard.

Matrix-matched calibration standards were generated for both linalool (in Chardonnay juice) and IBMP (in Pinot noir homogenate). These cultivars were selected because they did not have detectable concentrations of linalool or IBMP, respectively, by SPME-GC-MS. Figure 2 shows a representative DART-MS chronogram for IBMP and its internal standard spiked into Pinot noir homogenate, as well as a blank well. A minor interference at the quantifying transition for IBMP can be seen both the well containing only IBMP (Figure 2, left) and the blank well (Figure 2, middle). As with other SPME methods, the use of isotopically labeled standards during SPMESH sheet analyses is important for achieving good precision and accuracy during quantitative analyses to correct for discrimination during the extraction step [19,23]. Similar results were observed for linalool in Chardonnay (data not shown).

Previous work by our group on SPMESH sheet-DART-MS used only single stage Orbitrap-MS [18]. Linearity and limits of detection for the IBMP and linalool using MS-MS in comparison to our previous report is shown in Table 1. The limit of detection for IBMP was 10-fold lower in our current work (4 ng/L) as compared to previous work (39 ng/L) [18], an improvement that can credited to the use of MS-MS. Linalool detection limits (24 μg/L) were reduced by half to previous work (48 μg/L) [18], and were not improved with MS-MS methods (approximately 60 μg/L, data not shown).

### 3.2. Optimization of Grape Homogenate Preparation for IBMP Analysis

The new SPMESH sheet-DART-MS-MS method was used to quantify IBMP in grape homogenates produced from Bordeaux cultivars (Merlot, Cabernet Sauvignon) sourced from different vineyards in California, USA. These grape cultivars were selected because they often have IBMP at concentrations at or above sensory threshold (2 ng/L in water [4], 10–16 ng/L in wine [24,25]), and because IBMP is a useful quality marker for red wines produced from these grapes [26].

**Figure 3:**
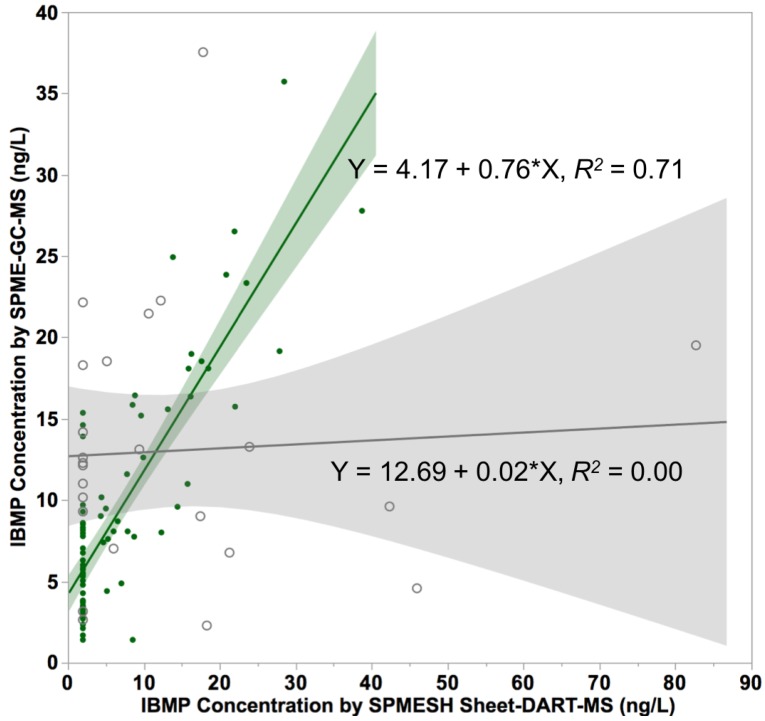
Initial (130 rpm, 50 °C, 30 min equilibration—gray open circles) and optimized (230 rpm, 70 °C, 1 h equilibration—green filled circles) correlation data for IBMP in Cabernet Sauvignon and Merlot grapes measured by SPMESH sheet-DART-MS, compared to SPME-GC-MS. Shaded areas represent 95% confidence intervals of the linear regression. Undetectable values were set to the square root of the limit of detection (IBMP = 2 ng/L for DART).

In our previous SPMESH sheet work on IBMP, grape macerate samples were equilibrated with the internal standard for 30 min at 50 °C with agitation prior to extraction [18]. In our previous work, we observed good recovery of IBMP in spiked Pinot noir samples which lacked native IBMP [18,19]. However, when we initially investigated Bordeaux cultivars with native detectable IBMP in our current work, we observed no correlation between SPMESH sheet-DART-MS-MS and the frequently-used SPME-GC-MS method (*R*^2^ = 0, Figure 3). We also observed high run-to-run variability for the SPMESH sheet method (CV = 63%). Using SPMESH, we also observed undetectable concentrations of IBMP in the supernatant (<4 ng/L) as compared to the homogenate (up to 24.9 ng/L, data not shown), suggesting that IBMP was not being sufficiently extracted from the skins into the liquid phase using our original conditions.

Previous work using SPME-GC-MS for quantitation of native IBMP (as opposed to spiked IBMP) in grape samples demonstrated that the IBMP/*d_3_*-IBMP ratio increases with longer SPME incubation times and/or higher incubation temperatures, eventually reaching an equilibrium [21]. These observations were reported to be due to the localization of IBMP in grape skins [27], such that native IBMP must first diffuse from the skins into the liquid phase before equilibrating with the deuterated standard and reaching the headspace SPME fiber.

To evaluate this possibility, we used a higher temperature equilibration step (230 RPM at 70 °C for 1 h) prior to SPMESH sheet extraction. These conditions were previously reported by Ryona, et al., as sufficient to reach IBMP/*d*_3_-IBMP equilibrium during SPME-GC-MS [21]. The improvement in SPMESH-DART-MS-MS vs. SPME-GC-MS correlation following introduction of this equilibration step is discussed in the next section.

One concern associated with using a high temperature incubation of grape samples is that thermal artifacts (isobaric interferences) could be generated. To evaluate this possibility, Pinot noir homogenate was incubated at 70 °C for 1 h while agitating at 230 RPM. We observed no detectable IBMP signal in both the control and incubated samples, indicating that thermal artifacts were unlikely (Figure 2). We also evaluated if IBMP signal measured by SPMESH-DART-MS-MS could be increased by addition of saturated NaCl solution, as is reported for SPME-GC-MS [28]. In our current work, we did not observe any increase in IBMP signal (data not shown; *p* > 0.05). The reason for the non-effect of NaCl addition on method sensitivity is unclear. In conventional coated fiber SPME analyses, the improvement in sensitivity with NaCl addition arises from a decrease in the solubility of the volatile in the NaCl enriched solution, which favors the partitioning of the volatile into the SPME fiber coating. Because SPMESH has a much larger PDMS volume than fiber-based SPME (4 × 4 cm square vs. 2 cm fiber), it is likely that SPMESH extraction is limited by the rate of mass transfer from the sample rather than the equilibrium partition coefficient, which would explain the lack of a “salting-out” effect in our current work.

### 3.3. Evaluation of SPMESH Sheet-DART-MS vs. SPME-GC-MS

#### 3.3.1. IBMP

**Figure 4:**
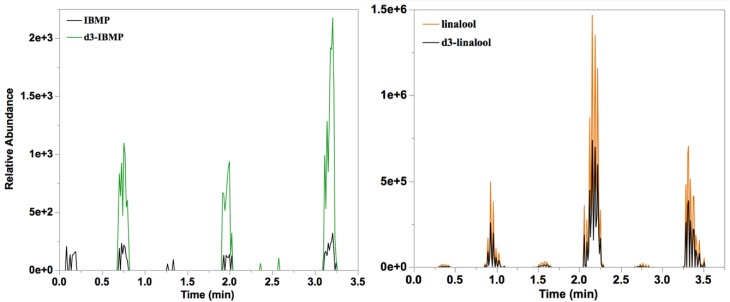
DART-Orbitrap-MS(-MS) chronograms of extracted ions for (**left**) IBMP 167 - > 124.0632 m/z and *d*_3_-IBMP 170 - > 127.0818 *m*/*z* of a Cabernet Sauvignon homogenate (average concentration = 20 ng/L by SPMESH sheet-DART-MS-MS; 24 ng/L by SPME-GC-MS) and (**right**) linalool 137.1326 *m*/*z* and *d*_3_-linalool 140.1512 *m*/*z* in Muscat of Alexandria juice (386 µg/L by SPMESH sheet-DART-MS; 353 µg/L by SPME-GC-MS). Samples were run in triplicate wells, with blank wells in between.

Representative chronograms for IBMP and its deuterated standard in homogenate under optimized conditions are shown in Figure 4. For evaluation of IBMP measurements, 86 samples of Cabernet Sauvignon and Merlot grapes were used. IBMP concentrations ranged from undetectable (<2 ng/L) to 28 ng/L by SPME-GC-MS and <4 ng/L to 38 ng/L by SPMESH sheet -DART-MS-MS. These values fall within the typical range of IBMP found in grapes during ripening: 100 ng/L to less than 10 ng/L [25,27]. Variation among detectable samples was 28% CV.

A good correlation (*R*^2^ = 0.71) was found between IBMP measured by SPMESH sheet-DART-MS and SPME-GC-MS (Figure 3). The correlation between SPMESH sheet and SPME methods was weaker than in our previous work (*R*^2^ = 0.97) comparing “one-shot” SPMESH cards with SPME-GC-MS [19]. However, the current work measured IBMP at native concentrations (<40 ng/L), as opposed to earlier work, which used IBMP spikes in the range of 200–1000 ng/L. Based on the regression equation, SPMESH sheet-DART-MS measured higher concentrations than SPME-GC-MS for high IBMP samples (slope = 0.76), but lower concentrations for low IBMP samples (*Y*-intercept = 4.1). The observation that SPME vs. SPMESH data has a slope <1 could arise from isobaric interferences on the SPMESH data set, but the reason for the non-zero intercept was unclear.

#### 3.3.2. Linalool

**Figure 5:**
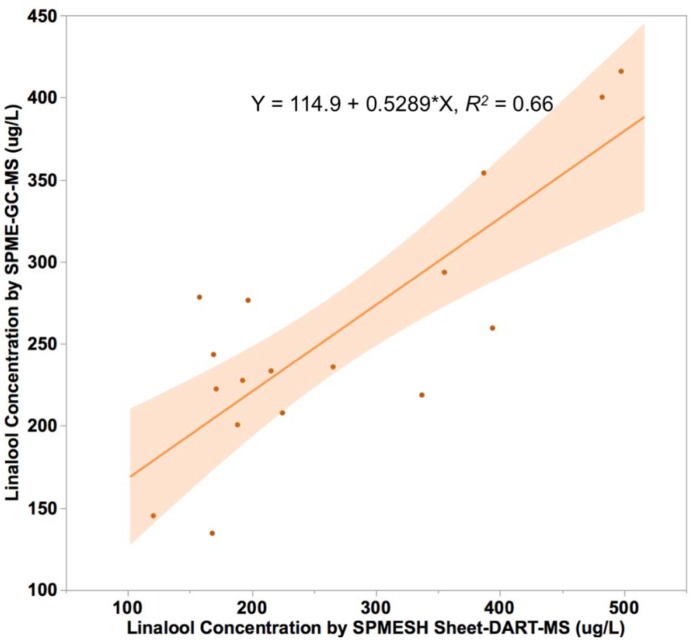
Correlation between linalool equivalents measured by SPMESH sheet-DART-MS, compared to SPME-GC-MS (solid phase microextraction-gas chromatography-mass spectrometry). Shaded area represents the 95% confidence interval of the linear regression.

Juice samples (*n* = 18) were prepared from Muscat-type grapes collected over several weeks of harvest. Linalool concentrations ranged from 135 to 415 μg/L by SPME-GC-MS and 120 to 497 μg/L by SPMESH sheet-DART-MS. These values are within the range for linalool found in Muscat grapes during ripening: approximately 60 to over 1000 μg/L from veraison to ripeness. A moderate correlation (*R*^2^ = 0.66) was observed between SPME-GC-MS and SPMESH sheet-DART-MS measurements (Figure 5). This correlation was comparable to our previous work using the “one-shot” SPMESH card and DART-MS [20]. Variation among detectable samples was 18% CV. As with the IBMP, SPMESH sheet-DART-MS measured higher concentrations than SPME-GC-MS for high linalool samples (slope = 0.52). We observed a similar phenomenon in our earlier SPMESH work [20] which at the time we credited to isobaric interferences from other monoterpenes. To evaluate this hypothesis, we re-evaluated the *m*/*z* = 137 traces from all SPME-GC-MS analyses. Although linalool was the major peak in all traces, we identified several other minor peaks corresponding to other well-known monoterpenes (e.g., linalool oxide, geraniol, α-terpeniol). We then summed the areas of all *m*/*z* = 137 peaks, or of the four major peaks (linalool, linalool oxide, geraniol, α-terpeniol) for each SPME-GC-MS analysis and plotted these values against SPMESH-DART-MS data. However, this resulted in worse correlation (*R*^2^ = 0.4 for all peaks, *R*^2^ = 0.46 for four major peaks; See Supplementary Figure S1). Thus, the correlation between SPMESH-DART-MS and SPME-GC-MS for linalool does not appear to be limited by interferences, although an alternative explanation is lacking at this time.

## 4. Conclusions

The SPMESH sheet approach yielded improvements in throughput and automation. Even with a 1 h equilibration, we could prepare, extract and analyze 24 samples from grapes to data in less than 3 h. This is a considerable improvement over SPME-GC-MS which, at best, could analyze 24 samples in 12 h not including sample preparation time. DART-MS analysis time can be further reduced by increasing positioning stage scanning speed, as demonstrated earlier [18]. This approach would result in a loss of sensitivity but may be appropriate for higher concentration targets like linalool in Muscat-type grapes, where concentrations are in excess of the methodological detection limit.

SPMESH sheet-DART-MS was used to measure two quality-related volatiles (linalool and IBMP) at native concentrations in a broad range of grape samples. Good correlations between SPMESH sheet-DART-MS and SPME-GC-MS were observed (R^2^ = 0.66–0.70). The SPMESH approach required 10-fold less instrument time than SPME-GC-MS. During SPMESH analyses, an offline heating and agitation step was necessary to equilibrate native IBMP with the labeled standard and achieve good equilibration. The use of tandem MS improved the detection limit for IBMP by an order of magnitude, to 4 ng/L, as compared to previous SPMESH-DART-MS work. This work demonstrates the suitability of SPMESH sheet-DART-MS as a rapid benchmarking platform for trace level IBMP and low-level linalool. The technique should also be amenable for adoption for analysis of volatile markers in either foods or beverages—especially those volatiles that are amenable to DART-MS analysis (e.g., can undergo proton-transfer reactions).

## Figures and Tables

**Table 1:** Figures of merit for IBMP and linalool in grape homogenate and juice, respectively.

-	IBMP (MS) ^a^	IBMP (MS-MS) ^b^	Linalool (MS) ^a^	Linalool (MS) ^b^
Calibration range	40.5–5000 ng/L	6.25–400 ng/L	405–50.000 μg/L	25–400 μg/L
*R* ^2^	0.96	0.99	0.99	0.99
LOD	39 ng/L	4 ng/L	48 μg/L	24 μg/L
LOQ	130 ng/L	12 ng/L	160 μg/L	72 μg/L
Average %CV ^c^	-	32	-	17

^a^ From Ref [19], ^b^ Using optimized conditions from the current paper. Preliminary evaluations of SPMESH-DART-MS-MS for linalool (approximate detection limit = ~60 µg/L) did not improve detection limits as compared to single stage MS, ^c^ Average %CV = (standard deviation/average) × 100.

## Supplementary Materials

The following are available online at https://www.mdpi.com/2304-8158/9/4/409/s1, Figure S1: Correlations plots of linalool by SPMESH-DART-MS vs. summed *m*/*z* = 137 peaks by SPME-GC-MS
