# Supplementary material for: Polymeric Sorbent Sheets Coupled to Direct Analysis in Real Time Mass Spectrometry for Trace-Level Volatile Analysis—A Multi-Vineyard Evaluation Study
Source: Foods. 2020 Apr 2;9(4):409. doi: 10.3390/foods9040409 (PMC7230477; doi:10.3390/foods9040409)
Supplement: Supplementary file 1 [file foods-09-00409-s001.pdf]

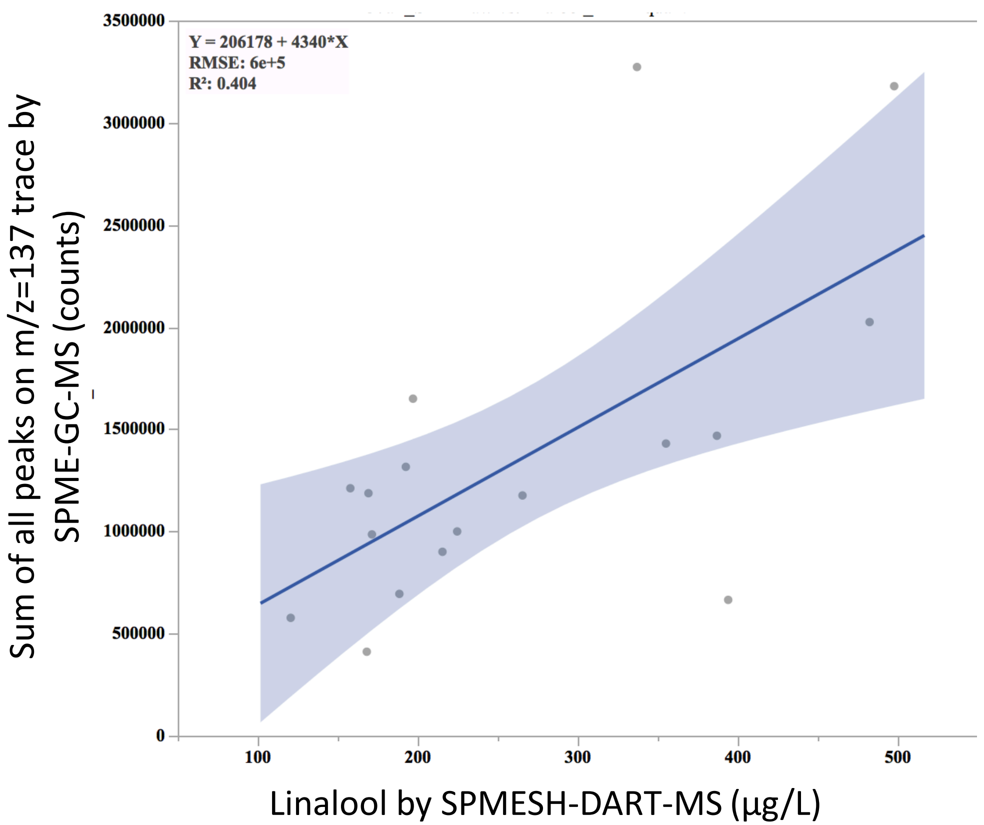

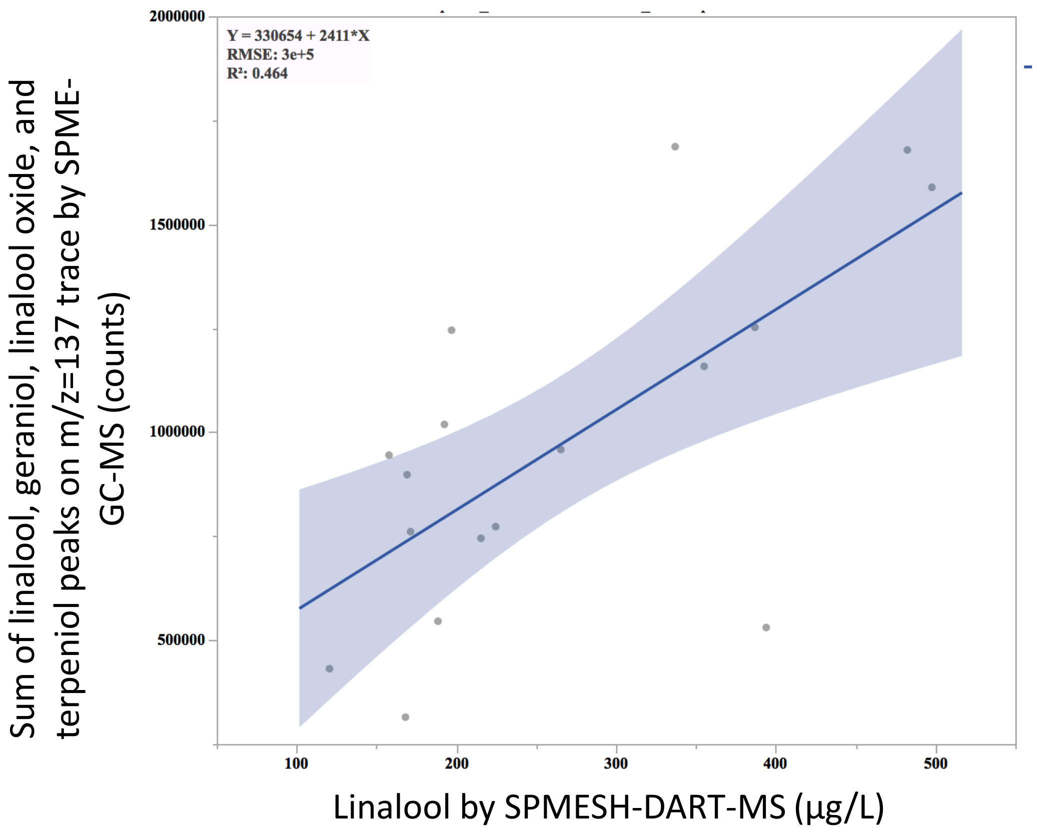

Supplementary Figure 1 – Plots of linalool measured across grape samples by SPMESH-DART-MS vs. the sum of all m/z = 137 peaks by SPME-GC-MS (left) and the sum of m/z=137 peaks for the four major monoterpenes (linalool, geraniol, linalool oxide, and alpha-terpeniol) by SPME-GC-MS (right).
